# Video Games for Well-Being: A Systematic Review on the Application of Computer Games for Cognitive and Emotional Training in the Adult Population

**DOI:** 10.3389/fpsyg.2018.02127

**Published:** 2018-11-07

**Authors:** Federica Pallavicini, Ambra Ferrari, Fabrizia Mantovani

**Affiliations:** Riccardo Massa Department of Human Sciences for Education, University of Milan Bicocca, Milan, Italy

**Keywords:** video games, computer games, cognitive training, emotional training, well-being

## Abstract

**Background:** Although several excellent reviews and meta-analyses have investigated the effect of video game trainings as tools to enhance well-being, most of them specifically focused on the effects of digital games on brain plasticity or cognitive decline in children and seniors. On the contrary, only one meta-analysis results to be focused on the adult population, and it is restricted to examining the effects of training with a particular genre of games (action video games) on cognitive skills of healthy adults.

**Objectives:** This systematic review was aimed to identify research evidences about the impact on cognitive [i.e., processing and reaction times (RTs), memory, task-switching/multitasking, and mental spatial rotation] and emotional skills of video games training in the healthy adult population.

**Methods:** A multi-component analysis of variables related to the study, the video games, and the outcomes of the training was made on the basis of important previous works. Databases used in the search were PsycINFO, Web of Science (Web of Knowledge), PubMed, and Scopus. The search string was: [(“Video Games” OR “Computer Games” OR “Interactive Gaming”)] AND [(“Cognition”) OR (“Cognitive”) OR (“Emotion”) OR (“Emotion Regulation”)] AND [“Training”].

**Results:** Thirty-five studies met the inclusion criteria and were further classified into the different analysis' variables. The majority of the retrieved studies used commercial video games, and action games in particular, which resulted to be the most commonly used, closely followed by puzzle games. Effect sizes for training with video games on cognitive skills in general ranged from 0.06 to 3.43: from 0.141 to 3.43 for processing and RTs, 0.06 to 1.82 for memory, 0.54 to 1.91 for task switching/multitasking, and 0.3 to 3.2 for mental spatial rotation; regarding video games for the training of emotional skills, effect sizes ranged from 0.201 to 3.01.

**Conclusion:** Overall, findings give evidences of benefits of video games training on cognitive and emotional skills in relation to the healthy adult population, especially on young adults. Efficacy has been demonstrated not only for non-commercial video games or commercial brain-training programs, but for commercial video games as well.

## Introduction

Over the last 40 years, video games have increasingly had a transformational impact on how people play and enjoy themselves, as well as on many more aspects of their lives (Yeh et al., 2001; Zyda, 2005; Boyle et al., 2012). Contrary to popular belief, which sees male children or teenagers as main targets of the gaming industry, the average player is instead 30 years old, and the entire gaming population is roughly equally divided into male and female players, therefore representing a daily activity for a consistent percentage of the adult population (Entertainment Software Assotiation, 2015). Thanks to the wide availability on the market, the affordable cost and the massive popularity, video games already represent crucial tools as a source of entertainment, and are soon expected to become critical also in another fields, including the mental health panorama (Granic et al., 2014; Jones et al., 2014).

While much of the early research on computer games focused on the negative impacts of playing digital games, particularly on the impact of playing violent entertainment games on aggression (e.g., Ferguson, 2007), and addiction (e.g., Gentile, 2009), gradually, scientific studies have also recognized the potential positive impact of video games on people's health (e.g., Anderson et al., 2010; Jones et al., 2014).

In recent decades, the field of computer gaming has increasingly developed toward serious purposes, and both commercial and non-commercial video games (i.e., developed *ad hoc* by researchers for the training of specific individuals' skills) have been tested by several studies. As early as in 1987, it was for the first time observed that famous commercial video games (i.e., *Donkey Kong* e *Pac-Man*) can have a positive effect on cognitive skills, improving the RTs of older adults (Clark et al., 1987). A few years later, in 1989, *Space Fortress*, the first non-commercial computer game designed by cognitive psychologists as a training and research tool (Donchin, 1989) was considered so successful that it was added to the training program of the Israeli Air Force. From that moment on, numerous video games have been developed with the specific purpose of changing patterns of behavior, and are often defined in literature as “serious games” (Zyda, 2005) as they use gaming features as the primary medium for serious purposes (Fleming et al., 2016).

Since these pioneering studies, numerous researches have investigated the potentiality of various video games, both commercial and non-commercial, mainly in relation with cognitive skills of seniors. For instance, it has been observed that the use of complex strategy video games can enhance cognitive flexibility, particularly in older adults (Stern et al., 2011). Furthermore, playing a commercial computer cognitive training program results in significant improvement in visuospatial working memory, visuospatial learning, and focused attention in healthy older adults (Peretz et al., 2011).

Besides being useful tools for the training of cognitive processes, various studies have demonstrated that video games offer a variety of positive emotion-triggering situations (e.g., Ryan et al., 2006; Russoniello et al., 2009; McGonigal, 2011), that may be of benefit during training of emotional skills, including self-regulation habits (Gabbiadini and Greitemeyer, 2017). For instance, puzzle video games such as *Tetris*, characterized by low cognitive loads and generally short time demands, are capable of positive effects on the players' mood, generating positive emotions and relaxation (Russoniello et al., 2009). Furthermore, by continuously providing new challenges, either it is switching from one level to another (e.g., *Portal 2*) or between different avatars (e.g., *World of Warcraft*), video games demand players to “unlearn” their previous strategies and flexibly adapt to new systems without experiencing frustration and anxiety (Granic et al., 2014).

Although several excellent reviews and meta-analyses have investigated the effect of video games training as tools for enhancing individuals well-being, in particular regarding cognitive and emotive enhancement (e.g., Boyle et al., 2016; Lumsden et al., 2016), most of them specifically focused on the effects of digital games on brain plasticity or cognitive decline in children and seniors (e.g., Lu et al., 2012; Lampit et al., 2014). Consonant findings regarding the positive relationship between video game training and benefits on various cognitive skills have been demonstrated by both behavioral studies (e.g., Baniqued et al., 2014) and meta-analytic studies (Toril et al., 2014) regarding both the aforementioned populations. On the contrary, only one meta-analysis focused on the adult population and it is restricted to examining the effects of training with a particular genre of games (action video games) on cognitive skills on healthy adults (Wang et al., 2016).

Despite this scarcity of focus on the adult population, the latter represents an extremely interesting and unique group, with very peculiar characteristics from a neurological and psychological point of view if compared to children and elders. As stated by Finch, the adult age, including both young adults (18–35 years old) and middle age adults (35–55 years old), plays an important role in the life-span development, and therefore very well deserves to be studied thoroughly (Finch, 2009). On the one hand, the effects of the so-called inverted U curve of neuroplasticity and cognitive performance starts to be evident during the adult age, especially the middle-age (Cao et al., 2014; Zhao et al., 2015). On the other, it is well known that the level of psychological stress perceived by adults is rather high, and it can result in important mental and health disorders (Kudielkaa et al., 2004).

Moreover, as the literature states, baseline individual differences regarding age can determine variations in training effectiveness (Jaeggi et al., 2011; Valkanova et al., 2014), and if it is safe to say that video games can have beneficial effects when included in a training (e.g., Baniqued et al., 2014; Toril et al., 2014), such effects might indeed vary based on age-specific aspects which therefore cannot be overlooked (Wang, 2017).

Consequently, in the current review, we will describe experimental studies that have been conducted between 2012 and 2017, with the aim to identify research evidences about the impact on cognitive and emotional skills of video games training in the adult population. Specifically, a multi-component analysis of variables related to the study, video games, and outcomes of training was made on the basis on important previous works (Connolly et al., 2012; Kueider et al., 2012; Boyle et al., 2016), which provide a useful framework for organizing the research along key variables.

## Methods

We followed the Preferred Reporting Items for Systematic Reviews and Meta-Analysis (PRISMA) guidelines (Moher et al., 2009).

### Search strategy

With the objective of providing an overview of the experimental studies that have been conducted to test the benefits of different categories of video games used as training tools of cognitive or emotional domains for the adult population, a computer-based search for relevant publications was performed in several databases. Databases used in the search were PsycINFO, Web of Science (Web of Knowledge), PubMed, and Scopus. The search string was: [(“Video Games” OR “Computer Games” OR “Interactive Gaming”)] AND [(“Cognition”) OR (“Cognitive”) OR (“Emotion”) OR (“Emotion Regulation”)] AND [”Training"].

### Selection of articles for inclusion in the review

To avoid the risk of bias, PRISMA recommendations for systematic literature analysis have been strictly followed (Moher et al., 2009). Two authors (Federica Pallavicini, Ambra Ferrari) independently selected paper abstracts and titles, analyzed the full papers that met the inclusion criteria, and resolved any disagreements through consensus. Selected papers have to: (a) include empirical evidences on the impact and outcomes of video game based training; (b) have been published during the last 5 years (namely from January 2012 to August 2017), in analogy with several other relevant previous works (i.e., Connolly et al., 2012; Boyle et al., 2016); (c) include participants within an age range of 18–59 years old; (d) only include samples of healthy participants, i.e., not suffering from any neurological disorder (e.g., traumatic brain injury), or psychiatric disorders according to DSM-5 Axis I (American Psychiatric Association, 2013); (e) be published on peer-reviewed journals.

### Coding of selected studies, video games, and training outcomes

The papers selected on the basis of the inclusion criteria were coded from the data extraction pro-forma that was developed by Connolly (Connolly et al., 2012), and subsequently modified by Boyle et al. (2016), adapting it to the specificity of this review and its area of interest. In particular, in this systematic review papers were coded with respect to:

- **Video Game Variables:** *The game category (*whether the game was commercial or non-commercial); *the game genre* (action games; driving-racing games; puzzle games; strategy games; simulation games; exergames; horror games; commercial brain training programs; arcade games; adventure games); *the platform for the game* (console, PC/laptop, or mobile gaming). First of all, the category of the game has been included to explore the effectiveness of several commercial titles, used “as-is” (without modifications), which in previous studies resulted to be effective for the cognitive training (e.g., Green and Bavelier, 2006; Dye et al., 2009). Furthermore, the categorization was included in order to analyze the efficacy of *ad hoc* developed games, about which an ongoing debate about their effectiveness still persists (e.g., Owen et al., 2010). Secondly, the classification of video game genres was considered because of the fact that, under many points of view, not all video games are equal and their effects strongly depend on specific characteristics of the game itself (Achtman et al., 2008; van Muijden et al., 2012). In addition, it has been reported that combinations between the neurological stage of the participants and the precise features of each video game produce unique results in a matter of benefits on mental skills (Ball et al., 2002; van Muijden et al., 2012). There is no standard accepted taxonomy of genre, although one of the most adopted is the Herz's system (Herz, 1997), while others studies seem to simply divide action games from any other kind, often defined as casual games as a whole (e.g., Baniqued et al., 2013, 2014). Here, we propose the above categorization, which resembles the present commercial classification as much as possible, defining ten different genres of commercial video games. Thirdly, new technologies such as mobile devices and online games have recently expanded the ways in which games have traditionally been played, their medium of delivery and the different platforms available. Platforms of delivery represent important information about video game training, primarily because they are the way in which the training itself can be accessed (Aker et al., 2016).
- **Variables Related to the Study:** *The sample* included in the study (sample size, mean age, or age range); *the research design* used (categorized as a Randomized Controlled Trial or Quasi Experimental); the *measures used for the assessment of outcomes* (self-report questionnaires, cognitive tests, fMRI, physiological data, etc.); *the duration of training* (duration, intensity, and the total amount of sessions); the *effects size of each training outcome*, reporting partial-eta squared (η^2^), with values closer to 1.0 indicating a stronger effect size, and Cohen's *d;* the calculation of range and mean value of effect sizes for each training outcome has been expressed as Cohen's *d*, applying the conversion formula when reported by the study in terms of partial-eta squared (η^2^) (Cohen, 1998); where not reported in the study, standardized Cohen's *d* effect sizes were derived following a computation formula: the one described in Dunlap et al. (1996) in order to calculate *d* from dependent *t*-tests; the computation formula by Thalheimer and Cook (2002) for ANOVAs with two distinct groups (*df* = 1); the calculation formula by Rosenthal and DiMatteo (2001) from χ^2^ (with one degree of freedom); otherwise, in cases where effect sizes could not be calculated because not reported in the study or because the necessary data to derive them through formulas were not present, *p*-value was reported instead ( e.g., Oei and Patterson, 2013; Wang et al., 2014; Chandra et al., 2016). The sample, study design, and measures of training outcomes have been included as relevant variables in analogy to what has been done in previous reviews (Boyle et al., 2012; Connolly et al., 2012), to facilitate the access to easily classified and comparable studies among the literature. An indication of mean age or age range has been provided in order to identify studies conducted on young vs. middle-aged adults. Training-related factors have also been considered, including the duration, intensity, and total amount of training sessions, as well as the effect sizes of the training outcomes, since they represent useful information about the characteristics and feasibility of the training itself (Hempel et al., 2004).
- **Video Game-Based Training Outcome Variables:** The selected papers have been divided into two *macro-categories*: cognition and emotion. Regarding cognition, authors identified five *domain-specific subcategories*, following the classification proposed by Kueider et al. (2012), partially adapted to the specificity of the results that emerged from the review, specifically: (1) *multiple domain*, namely trainings focused on more than one cognitive skill, such as trainings including reasoning, episodic memory, and perceptual speed as target skills at the same time; (2) *processing speed and reaction times* (RTs), i.e., respectively, the ability to quickly process information (Shanahan et al., 2006), and the amount of time needed to process and respond to a stimulus and is critical for handling information (Garrett, 2009)*;* (3) *memory*, defined as the ability to retain, store, and recall information (Baddeley and Hitch, 1974), including many different types of memory, such as episodic, short-term, visual and spatial working memory; (4) *task-switching/multitasking*, defined as a whole as attributes of control processes while switching from one task to another (Dove et al., 2000); (5) *mental spatial rotation*, that is the ability to mentally rotate an object (Shepard and Metzler, 1971). Such categorization has been chosen among many others proposed by literature (e.g., Sala and Gobet, 2016; Stanmore et al., 2017; Bediou et al., 2018), because of its particular adaptability to the search results at hand, and because of its effectiveness in defining precise sub-categories of cognitive skills.

## Results

### Papers identified by search terms

**Figure 1:**
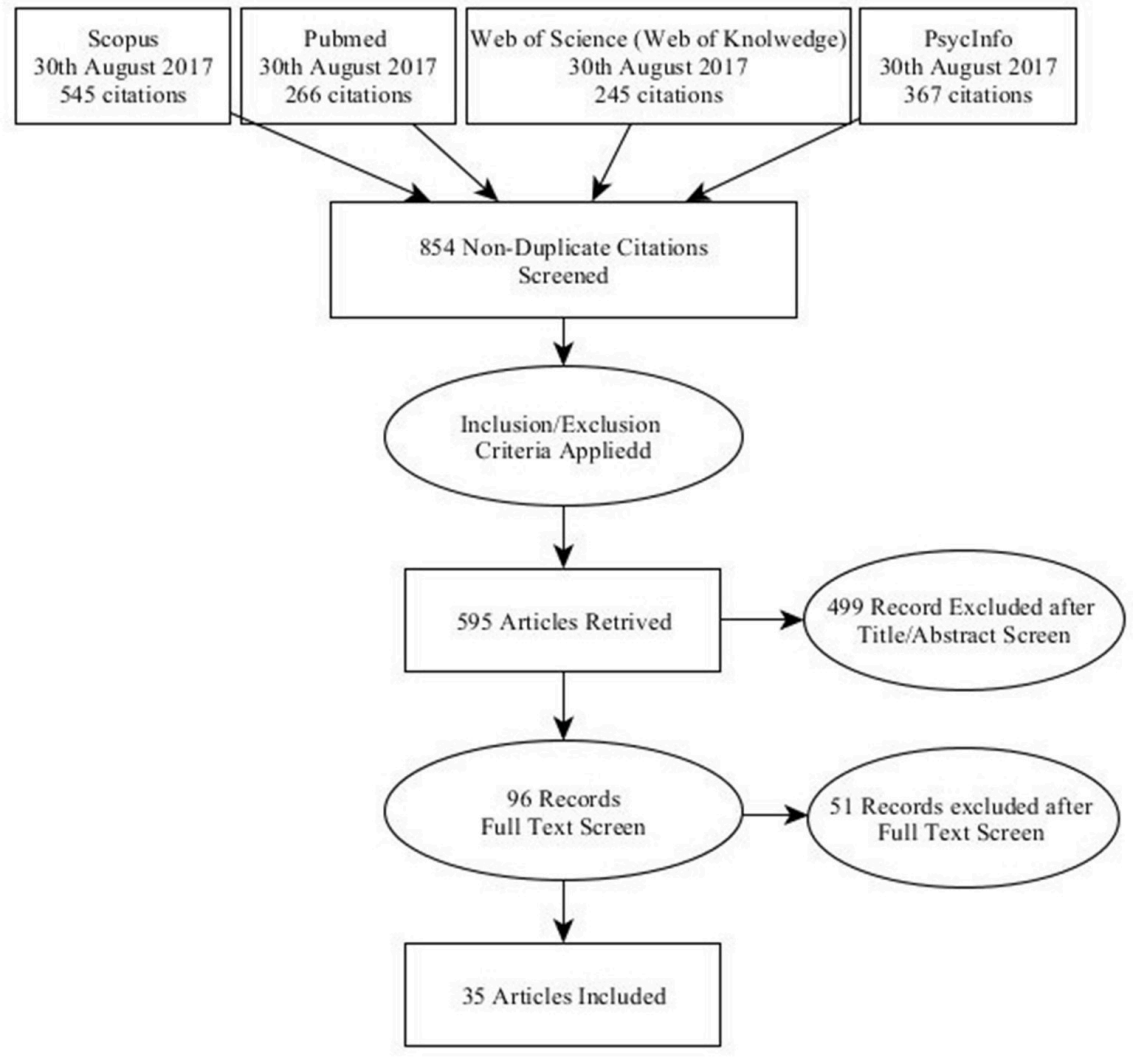
The flow chart of the systematic review.

A large number of papers (1,423) published in the time period between January 2012 and August 2017 was identified. As discussed in section Papers Selected Using our Inclusion Criteria, this set of papers was further screened, obtaining a set of 35 relevant papers (see Figure 1).

### Papers selected using our inclusion criteria

Applying the four inclusion criteria to these papers, 35 papers were identified (see Table 1). The largest number of papers was found in Scopus, followed by PsycINFO, Pubmed, and Web of Science (Web of Knowledge).

**Table 1:** Information about the video games variables of the selected studies.

**Study**	**Group**	**Video game**	**Video game category**	**Display**
Bailey and West, 2013	Video game group	Unreal tournament 3	Action game	Computer
	Video game group	Tetris	Puzzle game	Computer
	Control group	None	None	None
Baniqued et al., 2014	WMRS games group	Silversphere, Digital Switch, TwoThree, Sushi Go-Round	Puzzle games	Computer
	AWMR games group	Silversphere, Aengie Quest, Gude Balls, Block Drop	Puzzle games	Computer
	Active control group	Alphattack, Music Catch 2, Crashdown, Enigmata	Puzzle games	Computer
	Control group	None	None	None
Blacker et al., 2014	Action game group	Call of Duty: Modern Warfare	Action game	Computer
	Active control group	The Sims 3	Simulation game	Computer
Bouchard et al., 2012	Video game group	Left 4 Dead	Horror game	Computer
	Control group	None	None	None
Chandra et al., 2016	Video game group	Tom Clancy's Rainbow Six: Vegas 2	Action game	Computer
	Control group	None	None	None
Cherney et al., 2014	Nintendo Wii™ group	Segway Circuit mini-game	Exergame	Console (Nintendo Wii)
	Nintendo GameCube™ group	Crazy Taxi	Driving-racing game	Console (Nintendo GameCube)
	Control group	None	None	None
Choi and Lane, 2013	FPS game group	BeGone	Action game	Computer
	TPS game group	BeGone	Action game	Computer
	Control puzzle game group	Tetris	Puzzle game	Computer
	Control group	None	None	None
Clemenson and Stark, 2015	Video game group	Super Mario 3D World	Adventure game	Console (Wii U)
	Active control group	Angry Birds	Puzzle game	Console (Wii U)
	Control group	None	None	None
Colzato et al., 2013	Video game group	Half Life 2	Action game	Computer
Dennis and O'Toole, 2014	Video game group	The ABMT Personal Zen	Non-commercial	Mobile (iPod Touch)
	Active control group	The ABMT Personal Zen (Placebo)	Non-commercial	Mobile (iPod Touch)
Dennis-Tiwary et al., 2016	Video game group	The ABMT Personal Zen	Non-commercial	Mobile (iPod Touch)
	Active control group	The ABMT Personal Zen (Placebo)	Non-commercial	Mobile (iPod Touch)
Dominiak and Wiemeyer, 2016	Video game group	Dance paradise	Exergame	Console (Xbox 360)
	Video game group	Fable: The Journey	Action game	Console (Xbox 360)
	Control group	None	None	None
Green et al., 2012	Action video game group	Various	Action games	Various
	Active control group	Various	Various	Various
Hutchinson et al., 2016	Action video game group	Call of Duty: Modern Warfare 3	Action game	Console (Xbox 360)
	Action video game group	Call of Duty: Modern Warfare 3	Action game	Mobile (Nintendo DS)
	Cognitive video game group	Sight Training	Brain training game	Mobile (Nintendo DS)
	Control group	None	None	None
Kable et al., 2017	Cognitive video game group	Lumosity	Brain training game	Computer
	Active control group	Various	Puzzle games	Computer
Kühn et al., 2014	Video game group	Super Mario 64	Adventure game	Mobile (Nintendo DS)
	Control group	None	None	None
Lee et al., 2012	HVT group	Space Fortress	Non-commercial	Computer
	FET group	Space Fortress	Non-commercial	Computer
Li et al., 2016	Video game group	Mario Kart	Diving- racing game	Console (Nintendo Wii)
	Active control group	Roller Coaster Tycoon III	Simulation game	Computer
Looi et al., 2016	Real tDCS group	Mathematics video game	Non-commercial	Console (Xbox 360)
	Sham tDCS group	Mathematics video game	Non-commercial	Console (Xbox 360)
	Active control group	Mathematics video game	Non-commercial	Console (Xbox 360)
Mathewson et al., 2012	Video game group	Space Fortress	Non-commercial	Computer
Montani et al., 2014	Video game group	Labyrinth	Non-commercial	Computer
Naugle et al., 2014	Video game group	Wii Fit Boxing, Wii Fit Tennis, Wii Fit Step, Wii Fit Cycling	Exergame	Console (Wii Fit)
	Control group	None	None	None
Nikolaidis et al., 2014	Video game group	Space Fortress	Non-commercial	Computer
Nouchi et al., 2013	Video game group	Brain Age	Brain training game	Mobile (Nintendo DS)
	Active control group	Tetris	Puzzle game	Mobile (Nintendo DS)
Oei and Patterson, 2013	Action video game group	Hidden Expedition Everest	Puzzle game	Computer
	Hidden-object video game group	Bejeweled 2	Puzzle game	Computer
	Match-3 video game group	The Sims	Simulation game	Computer
	Memory matrix video game group	Memory Matrix 1.0	Brain training game	Computer
Oei and Patterson, 2014	Action video game group	Modern Combat	Action game	Mobile (smartphone)
	Puzzle video game group	Cut The Rope	Puzzle game	Mobile (smartphone)
	Real-time strategy video game group	Starfront Collision	Strategy game	Mobile (smartphone)
	Arcade video game group	Fruit Ninja	Arcade game	Mobile (smartphone)
Oei and Patterson, 2015	Metal gear solid video game group	Metal Gear Solid Touch	Action game	Mobile (smartphone)
	Modern combat video game group	Modern Combat: Sandstorm	Action game	Mobile (smartphone)
	Super sniper video game group	Super Sniper	Action game	Mobile (smartphone)
	Dear hunter video game group	Deer Hunter	Action game	Mobile (smartphone)
Parong et al., 2017	Video game group	Alien Game	Non-commercial	Computer
	Active control group	Bookworm	Puzzle game	Computer
Rolle et al., 2017	Video game group (young)	Pocket Bowling 3D	Simulation game	Mobile (smartphone)
	Control group (young)	None	None	None
	Video game group (old)	Pocket Bowling 3D	Simulation game	Mobile (smartphone)
	Control group (old)	None	None	None
Schubert et al., 2015	Video game group	Medal of Honor	Action game	Computer
	Video game group	Tetris	Puzzle game	Computer
	Control group	None	None	None
Shute et al., 2015	Video game group	Portal 2	Puzzle game	Computer
	Active control group	Lumosity	Brain training game	Computer
Stroud and Whitbourne, 2015	Video game group	Bejeweled Blitz	Puzzle game	Computer
	Video game group (10 rounds)	Bejeweled Blitz	Puzzle game	Computer
	Video game group (30 rounds)	Bejeweled Blitz	Puzzle game	Computer
	Control group	None	None	None
van Ravenzwaaij et al., 2014	Video game group	Unreal Tournament 2004	Action game	Computer
	Active control group	The Sims 2	Simulation game	Computer
	Control group	None	None	None
Wang et al., 2014	Video game VG players group	Call of Duty 2	Action game	Computer
	Video game non-VG players group	Call of Duty 2	Action game	Computer
Wu and Spence, 2013	Video game group	Medal of Honor: Pacific Assault	Action game	Computer
	Video game group	Need for Speed: Most Wanted	Driving-racing game	Computer
	Video game group	Ballance	Puzzle game	Computer

### Analysis of game variables

#### Video games category

Considering the entirety of the studies, 42 commercial video games and 7 non-commercial video games have been tested as training tools for cognitive or emotional skills. As for video games used for cognitive enhancement specifically, a total of 38 commercial video games and 6 non-commercial video games have been adopted; concerning emotional enhancement, instead, 4 commercial games and 1 non-commercial game have been used as training tools in the studies included in this review.

#### Video games genres

Among the studies included in this systematic review, the genre of commercial games was very varied, with action games (15) being the most used, followed by puzzle games (8), brain training games (5), exergames, and driving-racing games (3 for each category), simulation, driving racing games and exergames (3 games each), adventure games (2 games for each genre), and, finally, strategy games, arcade games, and horror games (1 game for each genre). Regarding training of cognitive skills specifically, among commercial games, the genre was very varied, with action games (14) and puzzle games (7) being the most used, followed by brain training games (5), simulation and driving-racing games (3 games), exergames and adventure games (2 games each), and, finally, strategy games and arcade games (1 game for each genre). As for emotional training, only 1 study adopted a non-commercial video games, while a variety of commercial video games were used (1 horror game, 1 action game, 1 puzzle game, and 1 exergame).

#### Platform/delivery

Considering the retrieved studies, games delivered via PC or laptop were the most popular in all categories (20 studies), followed by mobile (8 studies) and console (7 studies). Regarding cognitive training, 18 video games delivered via PC were used, 6 via console, 6 via mobile. As for emotional training, 2 video games were delivered via PC, 2 via console, and 1 via mobile.

### Analysis of variables related to the study

#### Sample

The mean number of participants included in the emerged studies was 54.4 (cognition: *M* = 56.1; emotion: *M* = 42.8), ranging between 5 (Chandra et al., 2016) and 209 (Baniqued et al., 2013). The samples' mean age, instead, was 24.2 (cognition: *M* = 23.8; emotion: *M* = 27.7).

#### Study design

In general, 28 studies included in the review have use a randomized control trial (RCT), while 7 studies have used a quasi-experimental design. The RCT was the design of choice of 24 studies related to cognitive training (e.g., Hutchinson et al., 2016; Looi et al., 2016). A quasi-experimental design was instead adopted in six studies directed at the evaluation of cognitive trainings based on video games (Mathewson et al., 2012; Montani et al., 2014). As for emotional training, four studies followed a RCT design (e.g., Bouchard et al., 2012), while 1 a quasi-experimental design (Naugle et al., 2014).

#### Duration of the training

The length of the trainings proposed by studies included in this systematic review resulted to be rather heterogeneous, both in the number of sessions and in the number of weeks. In particular, the mean number of sessions was 10.1, ranging from 1 to 60 sessions, while the mean number of hours played was 13.5, ranging between 10 min and 50 h. As for cognitive training, a minimum of one session (e.g., Colzato et al., 2013; Cherney et al., 2014), and a maximum of 60 sessions (Kühn et al., 2014). The number of hours spent playing the different video games differed from study to study as well: from several minutes (Stroud and Whitbourne, 2015) to up to 50 h (Green et al., 2012; Chandra et al., 2016). As for emotional training, the minimum number of sessions was 1 as well, while the maximum was 10 (Bailey and West, 2013); the minimum time spent playing was of 25 min (Dennis and O'Toole, 2014; Dennis-Tiwary et al., 2016), and the maximum was 10 h (Bailey and West, 2013).

#### Measures used for the assessment of outcomes

The measures of the training outcome adopted in the studies included in this systematic review predictably have largely been constituted by cognitive tests, for a total of 33 studies, 30 related to cognitive training (e.g., Baniqued et al., 2014), and 3 to emotional training (e.g., Bailey and West, 2013). Nonetheless, numerous studies (19) have included self-administered psychological questionnaires: 14 aimed at cognitive training (e.g., Chandra et al., 2016), and 5 to emotional training (e.g., Dennis and O'Toole, 2014), while physiological measures were used in a total of 2 studies, both emotional trainings (e.g., Bouchard et al., 2012). fMRI-based assessments were instead used to measure the outcomes of cognitive trainings in two studies (e.g., Nikolaidis et al., 2014) and EEG assessments were used in a total of three studies (1) related to cognitive training (i.e., Mathewson et al., 2012), and (2) to emotional training (e.g., Bailey and West, 2013).

### Analysis of video game training outcomes

#### Cognition

Thirty studies used cognitive domain-specific training programs including memory, task-switching/multitasking and mental spatial rotation. Across all cognitive trainings, the effect sizes' (Cohen's d) range was 0.141–3.43 for processing and RTs (*M* = 1.18), 0.06–1.82 for memory (*M* = 0.667), 0.54–1.91 for task-switching/multitasking (*M* = 1.11), and 0.3–3.2 for mental spatial rotation (*M* = 1.5).

- **Multiple domains (13 studies):** Because of the wide literature consensus about the little to non-transferability of cognitive training effects to untrained skills (Rebok et al., 2007), a rather high number of retrieved studies aimed at the enhancement of multiple cognitive domains with a single training, with the objective of deepening our knowledge about generalizability across domains. Training with action video games has been reported to enhance processing speed and RTs (Oei and Patterson, 2013, 2015; Schubert et al., 2015; Chandra et al., 2016), but no effect on spatial (Oei and Patterson, 2015) nor visual (Schubert et al., 2015; Chandra et al., 2016) working memory has been reported. Concerning other categories of commercial video games, training with puzzle games was shown to improve task switching skills and inhibitory control, but not visual and spatial working memory, episodic memory or perceptual speed (Baniqued et al., 2014). Spatial working memory, as well as RTs, improved after training with a simulation game (Rolle et al., 2017). Better RTs have also been reported after training with FPS games (i.e., first person shooter games, in which the player shoots at targets while witnessing the scene as through the eyes of the character they are controlling), which also seemed to have positive effects on processing speed, but not on mental spatial rotation skills (Choi and Lane, 2013). Moreover, it was reported that video game training with an adventure game can augment gray matter in brain areas crucial for spatial navigation and visual working memory, along with evidence for behavioral changes of navigation strategy (Kühn et al., 2014). As far as brain training games are concerned, studies confirmed that this genre of video games can improve task-switching, short-term memory, RTs and processing speed more heavily compared to a puzzle game (Nouchi et al., 2013). Nonetheless, two different studies did not highlight any advantage of puzzle games over other video game genres in enhancing cognitive skills such as mental spatial rotation (Shute et al., 2015), nor for cognitive performance on other domains (e.g., task-switching, visual, and spatial working memory) (Kable et al., 2017). Lastly, a non-commercial game, *Space Fortress*, was proven to be effective as a training for visual working memory (Lee et al., 2012), and alpha and delta EEG oscillations during game play of this particular video game were shown to predict learning and improvements in such cognitive skill, while no similar effects were found on task-switching/multitasking skills (Mathewson et al., 2012).
- **Processing speed and reaction times (8 studies):** Studies reported that action games (Green et al., 2012; Wang et al., 2014), FPS games (Colzato et al., 2013; Hutchinson et al., 2016), adventure (Li et al., 2016), and puzzle games (Stroud and Whitbourne, 2015) can be considered effective training tools for processing speed and RTs. Moreover, in a study comparing the effectiveness of various genres of commercial video games, action and driving-racing games were proven to decrease RTs and processing speed more effectively than a puzzle game (Wu and Spence, 2013). Only one study did not report any benefit of commercial video games over these particular skills (van Ravenzwaaij et al., 2014).
- **Memory (4 studies):** Effective trainings of visual working memory have been carried out with an action game (Blacker et al., 2014) as well as with an adventure game (Clemenson and Stark, 2015). Concerning non-commercial video games, a mathematics video game training was shown to be effective on short-term and visual working memory (Looi et al., 2016). Furthermore, individual differences in the post-minus-pre changes in activation of regions implicated in visual working memory during gameplay of an *ad hoc* developed game (*Space Fortress*) have been reported to predict performance changes in an untrained working memory task (Nikolaidis et al., 2014).
- **Task-switching/multitasking (3 studies):** The cost of dual tasking, as well as the cost of task switching, decreased after training with a custom-made video game (Montani et al., 2014). Moreover, a training based on an *ad hoc* developed game lead to significantly better performance on cognitive shifting tests after playing for 2 h over four sessions (i.e., reaching a high level in the game) (Parong et al., 2017). The same results, in fact, were not obtained if participants were asked to play for only 1 h over two sessions (Parong et al., 2017). Furthermore, playing commercial puzzle games improved task-switching ability (Oei and Patterson, 2014).
- **Mental spatial rotation (2 studies):** Enhancement of mental spatial rotation abilities was reported after training with commercial exergames and driving-racing games, with a greater advance for women (Cherney et al., 2014). In contrast, no improvement was observed after training with other commercial games (one exergame and several action games), probably because of the limited number of participants (Dominiak and Wiemeyer, 2016).

#### Emotion

Five studies tested video games as tools for training emotional skills (Table 2). Across all these training programs, the effect sizes' range (Cohen's *d*) was 0.201–3.01 (*M* = 0.897). First of all, playing a commercial action game resulted in brain changes related to the emotion processing of facial expressions, with a reduction in the allocation of attention to happy faces, suggesting that caution should be exercised when using action video games to modify visual processing (Bailey and West, 2013). Moreover, playing exergames at a self-selected intensity has been reported to positively influence emotional responses (enjoyment, changes in positive and negative affects) (Naugle et al., 2014). Interestingly, commercial video games have also been tested as a tool to provide interactive Stress Management Training (SMT) programs, mainly used for decreasing levels of perceived stress and negative effects. In particular, training with a commercial horror video game combined with arousal reduction strategies (e.g., exposure to stressful scenarios, traditional biofeedback techniques) has shown efficacy in increasing resilience to stress in soldiers, as observed through analyses of salivary cortisol level conducted along the training (Bouchard et al., 2012). Regarding non-commercial video games, training with an *ad hoc* non-commercial video game has been shown to help trait-anxious adult people handle emotional and physiological responses to stressors (Dennis and O'Toole, 2014), as well as improve behavioral performance in an anxiety-related stress task among female participants (Dennis-Tiwary et al., 2016).

**Table 2:** Information about the selected studies on video games for emotional training.

**Study**	**Sample**	**Age**	**Study design**	**Conditions**	**Time spent playing**	**Measures**	**Main outcomes**	**Effect sizes**
Bailey and West, 2013	31	18–45 years old	Randomized controlled trial	Three-conditions: – Action video game group – Non-action video game group – No contact control group	Ten sessions (1 h each) across 10 consecutive days	Pre and post training: – The Emotion Search Task while ERPs (Event Related Potentials) are recorded	Playing an action game resulted in a change in the affective information processing, with an the an increase in the amplitude of the ERPs over the right frontal and posterior regions that was similar for angry, happy, and neutral faces	– For the action group, the latency of the P3 ERP component was shorter for the post-test than for the pre (η^2^ = 0.44); for the no-contact and non-action group, the main effect of occasion was not significant (η^2^ = 0.17)
Bouchard et al., 2012	41	24.9 ± 3.9 years old	Randomized Controlled Trial	Two conditions: – Video game group – Training-as-usual control group	Three sessions of 30-min (one per day for 3 days)	Pre and post training: – Concentration of salivary cortisol and heart rate – Perceived sense to control stress	Decrease of stress level after the training with the video game	– When comparing efficacy of the ImPACT program on the main measure of stress, there was a significant difference in cortisol response (η^2^ = 0.17) documenting that the program was effective in better controlling stress than training as usual – The ImPACT program had a significant positive impact on stress levels measured thought heart rate (η^2^ = 0.11) during the apprehension phases – Significant interaction Time × condition on self-reported perceived stress (η^2^ = 0.31), with ImPACT program contributing for the greater decrease
Dennis and O'Toole, 2014	78	17–50 years old	Randomized Controlled Trial	Four conditions: – Video game group short training – Active control group short training – Video game group long training – Active control group long training	Single session (45 min for the long training condition and 25 for the short training condition)	Pre and post training: – Tries social stress test (TSST) (stress reactivity) – Baseline mood questionnaire – Threat bias (dot-probe task)	Evidence that an alternative delivery strategy for ABMT— a gamified mobile app — shows transfer of benefits to independent, untrained lab-based measures of anxiety and stress reactivity after a single session of training. The long but not the short active training condition reduced the core cognitive process implicated in ABMT (threat bias)	– Threat bias: significant main effect of Training Condition on attentional bias (η^2^ = 0.13), and disengagement (η^2^ = 0.19); the effect for vigilance did not reach significance (η^2^ = 0.01) – Significant main effect of training condition on self-reported anxiety (η^2^ = 0.10)
Dennis-Tiwary et al., 2016	42	18-38 years old	Randomized controlled trial	Two conditions: – Video game group – Active control group	One session (25min of total gameplay with two 10-min breaks)	Pre and post training: – State-trait anxiety inventory (STAI) – Threat bias (Dot-probe task) while recording EEG activity –Trier social stress test (TSST)	Improved behavioral performance during the anxiety-related stress task among female participants only	The main effect of Training Group did not reach significance for observed behavioral performance (η^2^ = 0.02); there was a significant interaction between training group and gender for observed behavioral performance (η^2^ = 0.13): behavioral performance was better for the ABMT vs. placebo training (PT) condition, but only for females (*p* = 0.02); for the PT condition only, males had better performance compared to females (*p* = 0.02) – The interaction between Training Group and Gender was significant for N2 amplitudes to threat vs. non-threat (η^2^ = 0.11): post-training N2 amplitudes to threat vs. non-threat were greater following ABMT vs. PT, but only for males (*p* = 0.02); for the PT condition only, post-training N2 amplitudes to threat vs. non-threat were greater for females vs. males (*p* = 0.02) – No other effects reached significance
Naugle et al., 2014	22	20.5 ± 1.1 years old	Quasi-experimental	Two conditions: – Video game group – Traditional physical activity group	Three sessions, 2 exercise activities per session for 20 min each	Pre and post training: – Heart rate – Enjoyment – Positive and negative affect scale (PANAS)	Wii boxing and Wii tennis elicited the highest levels of enjoyment and produced an increase in positive emotions after game play	– Significant effect of Activity on average heart rate (*d* = 3.01); however, the main effect was superseded by a significant Activity × Group interaction (*d* = 1.07) – Significant effect of Activity for level of enjoyment (*d* = 1.08); follow-up tests indicated that the participants enjoyed playing Wii tennis significantly more than they did all the other exercise activities except for Wii boxing – Significant Activity × Time interaction (*d* = 0.74): the *post hoc* tests showed that positive affect significantly (a) decreased after walking and running on the treadmill, and (b) increased after playing Wii tennis and Wii boxing

## Discussion

In the present systematic review, we examine experimental studies that have been conducted with the aim to identify research evidences about the impact on cognitive and emotional skills of video games training in the healthy adult population. The large number of papers (1,423) identified using our search terms confirmed that there has been a surge of interest in the use of games for the aforementioned specific population, following the tendency already registered about elders (e.g., Lampit et al., 2014), and young people (e.g., Gomes et al., 2015). After the application of the inclusion criteria, 35 papers were finally included and described on the basis of important previous works, which provide a useful framework for organizing the research along key variables (Connolly et al., 2012; Kueider et al., 2012; Boyle et al., 2016).

With respect to *video game variables*, starting from the *games' category*, efficacy was demonstrated not only for non-commercial video games or commercial brain-training programs, but for commercial off-the-shelf video games as well. Interesting cases regard Tetris, which resulted to be more effective than a commercial brain training program (i.e., *Brain Age*) in improving cognitive skills such as short-term memory and processing speed (Nouchi et al., 2013), and *Portal 2*, that has proven to be effective in improving skills such as problem solving even more effectively than a brain training program specifically developed for this purpose (i.e., *Lumosity)* (Shute et al., 2015). The fact that not only *ad hoc* non-commercial games, but also commercial video games can be useful for training cognitive and emotional capacities, if confirmed, appears to be very interesting, as it opens the possibility to use commercial titles for the training of cognitive and emotional abilities in the adult population. This could mean increasing adherence to training, keeping the trainee engaged with an effective feedback system (Cowley et al., 2008), and enhancing the accessibility of training programs in terms of costs and ease of access to treatment, since it would be sufficient to simply have a console or another gaming device.

As for the distribution of *game genre*, considering only commercial games, in the emotional training sector no genre prevalence is recorded, while in cognitive training action games are the most commonly used, followed by puzzle games, and by brain training games. Such result should not be considered surprising, as previous literature indicates action games as the class of video games which has been scientifically assessed for the longest time (e.g., Adachi and Willoughby, 2011), similarly to puzzle games (e.g., Carvalho et al., 2012), and brain training games (e.g., Owen et al., 2010).

Results showed that the *delivery platform* of choice for more than half of the included studies was the PC, distantly followed by games delivered via consoles or via mobile. This distribution is valid for both commercial and non-commercial games, which seems to be a rather interesting fact and various reasons behind this consistency of distribution can be hypothesized. Future studies should better investigate especially mobile training, which, because of its potential ubiquity, its low costs, and its potentially real-time use, could offer unique advantages over traditional tools such as PCs.

Regarding the *variables related to the studies*, namely the *sample characteristics*, the results of this systematic review showed that the majority of studies have been conducted on young adults (18–35 years) rather than middle-aged adults (35–55 years). A possible explanation of this tendency could be linked to the fact that many studies have enlisted college students as participants, for a matter of simplicity of recruitment. However, it is important to note that the differentiation between young and middle-aged adults can be particularly relevant. As it is reported by scientific literature, in fact, the effects of the so-called inverted U curve of neuroplasticity and cognitive performance and of the perceived stress starts to be evident during the middle-age (Cao et al., 2014; Zhao et al., 2015). Moreover, strong differences in terms of knowledge and use of video games characterize these two age ranges. For these reasons, future studies should better investigate differences and analogies between young and middle-aged adults, for instance to identify in which life-span moment a game-based cognitive or emotional treatment would potentially be more effective.

Secondly, regarding *the experimental design* adopted in the studies, results show that in the majority of cases studies were conducted using a RCT design. This seems to be linked to the need for evidences of well-controlled studies, differently from previous studies in which less strong methods (e.g., survey, correlational design) were used. It will be important for future studies to continue using this type of experimental design, which is considered as the most reliable empirical design in order to prove a treatment's effectiveness, minimizing the impact of confounding variables (Levin, 2007).

The *measures of outcome of the training* adopted in the studies included in this systematic review predictably have largely been constituted by cognitive tests (e.g., Blacker et al., 2014). Nonetheless, numerous studies have included self-administered psychological questionnaires (e.g., Nouchi et al., 2013), physiological measures (e.g., Naugle et al., 2014), EEG-based assessment measures (e.g., Dennis-Tiwary et al., 2016), and fMRI-based assessments measures (e.g., Kable et al., 2017), which seem to be more reliable in assessing change over time, therefore an openness to such ways of assessment is desirable in a perspective of empirical evidence.

The *length of the training programs* proposed by studies included in this systematic review resulted to be rather heterogeneous, both in the number of sessions and in the number of weeks: from a minimum of one session (e.g., Colzato et al., 2013; Cherney et al., 2014) to a maximum of 60 sessions (Kühn et al., 2014), and with gameplay time ranging from 10 min to 50 h (Green et al., 2012; Chandra et al., 2016). Since the duration and intensity of training has been reported to be a relevant variable, as it has a rather important impact on the accessibility and feasibility of the training itself (Hempel et al., 2004), future studies should address in detail such aspects of the training, for instance comparing the effectiveness of shorter trainings to longer ones in order to identify the minimum number of sessions to obtain an effective program.

Finally, regarding the *training outcome*, based on this review, video games appear to hold promise for improving both cognitive and emotional skills in the healthy adult population. Empirical evidences were identified for all the training outcomes (i.e., cognition: multiple domain, processing speed and RTs, memory, task-switching/multitasking, mental spatial rotation; emotion).

Effect sizes (Cohen's *d*) for cognitive training, in general, ranged from 0.06 to 3.43: in particular from 0.141 to 3.43 for processing and RTs, 0.06 to 1.82 for memory, 0.54 to 1.91 for task-switching/multitasking, and 0.3 to 3.2 for mental spatial rotation (Table S1). Effect sizes reported in this systematic review are comparable to those reported for video game interventions aimed at enhancing cognitive skills of senior populations (Kueider et al., 2012; Lampit et al., 2014). For instance, a systematic review of a computerized cognitive training with older adults reported a range standardized pre-post training gain from 0.09 to 1.70 after the video game intervention, which appears to be similar to the values emerged from the traditional (0.06–6.32) or computerized (0.19–7.14) trainings (Kueider et al., 2012).

Based on the studies reviewed, the largest impact of video game trainings for cognitive skills was found on processing speed and RTs, as these cognitive domains presented the larger effect sizes. In particular, it has been observed that training with action games (Green et al., 2012; Wang et al., 2014), FPS games (Colzato et al., 2013; Hutchinson et al., 2016), adventure (Li et al., 2016), and puzzle games (Stroud and Whitbourne, 2015) can enhance these skills in healthy adults. In only one case no benefits have been reported over these particular skills after training with commercial video games (van Ravenzwaaij et al., 2014). The possibility to train processing speed and RTs with video games, especially with action video games, represents one of the largest interests of video game and cognitive training literature in spite of mixed results about its effectiveness (e.g., Dye et al., 2009; Wang et al., 2016), therefore further investigation is surely needed. For instance, action video game novices assigned to action video game training show faster visual information processing according to one study (Castel et al., 2005), while no improvement has been reported for seniors involved in a brief training (Seçer and Satyen, 2014).

Results were generally positive across studies on training of memory as well. In particular, improvements in visual and spatial working memory have been observed after training with an action game (e.g., Blacker et al., 2014), an adventure game (Clemenson and Stark, 2015), and a non-commercial game (Looi et al., 2016). Concerning other forms of memory, a positive effect of an adventure game-based training on mnemonic discrimination was reported in one study (Clemenson and Stark, 2015), while improvements in short term memory skills have been noticed after a brain training program (Nouchi et al., 2013). On the contrary, no positive effects on episodic memory nor on visual and spatial working memory have been reported after training with puzzle games (Baniqued et al., 2014). What emerged from the studies included in this review appears to be in line with previous evidences concerning the possibility to effectively use video games to enhance the memory skills of young and older populations, in particular regarding visual and spatial working memory (e.g., Wilms et al., 2013; Toril et al., 2014). It is nonetheless important to highlight the fact that, in this systematic review and in previous literature, the efficacy (or the ineffectiveness) of each training seems to differ on the basis of the specific game genre, as well as of the sample characteristics (e.g., Baniqued et al., 2013; Oei and Patterson, 2015; Chandra et al., 2016). Future studies are therefore necessary in order to better investigate the role of video games in such sense.

Regarding mental spatial rotation, even though the effect sizes are averagely high, only two studies have been included in this review, therefore results should be considered in the context of such numerical limitation. From what emerged from this systematic review, an enhancement of mental spatial rotation abilities was reported after training with commercial exergames and driving-racing games, with a greater advance for women (Cherney et al., 2014), while no improvement was observed after training with other commercial games (one exergame and several action games) (Dominiak and Wiemeyer, 2016). Since early findings in this research field have reported evidences supporting an enhanced performance in spatial relations after video game training in elders (e.g., Maillot et al., 2012) and children (Subrahmanyam and Greenfield, 1994), future studies should deeply verify the possible usefulness of video games as training of such cognitive skill in adults specifically.

As for task-switching/multitasking, in spite of high effect sizes suggesting the effectiveness of video game trainings in such sense, it is once again important to underline the limited number of considered studies (three). According to the included studies, the cost of dual tasking and the cost of task-switching decreased after training with a commercial puzzle game (Oei and Patterson, 2014), as well as with a custom-made video game (Montani et al., 2014; Parong et al., 2017). The use of video games for such purpose, because of their own nature of requiring complex planning and strategizing, appears to be rather significant, as it could potentially allow training or rehabilitation of these cognitive skills (e.g., Boot et al., 2008). Literature, nonetheless, still presents mixed results, not always positive (e.g., Green et al., 2012), and for this reason future studies providing an in-depth analysis are still necessary.

Finally, regarding video games for the training of emotional skills, effect sizes ranged from 0.201 to 3.01. Despite the generally high values, it is currently impossible to compare them with results emerged from other systematic reviews or meta-analyses concerning the same topic, as the few works around the subject do not provide any information about effect sizes (e.g., Villani et al., 2018). The studies included in this review provide evidences suggesting that non-commercial video games (Dennis and O'Toole, 2014; Dennis-Tiwary et al., 2016) and commercial video games (exergames and horror games) can be effective in inducing positive emotions and in reducing individual levels of stress in healthy adults (Bouchard et al., 2012; Naugle et al., 2014). From this review, it appears that the number of studies conducted about this kind of training is smaller than the amount of studies related to cognitive training. This fact is rather curious, because the video games' intrinsic characteristics of being motivating, engaging, and easily accessible (Granic et al., 2014), make computer games potentially useful tools in order to better the individuals' emotion regulation. Future studies will be fundamental in order to explore the potentiality of video games as emotional training tools, and to identify the most effective game genres for this purpose, examining potentially interesting genres that have not been investigated yet (e.g., affective gaming, virtual reality-based gaming).

### Limitations

As with all literature reviews, the current review does not claim to be comprehensive, but summarizes the current research on video games for the cognitive and the emotional training in the adult population based on specific key words used in the search string, the database included and the time period of the review. Moreover, in this review we based our choice of categories on a specific model (Connolly et al., 2012; Kueider et al., 2012; Boyle et al., 2016), however the level of specificity and distinctiveness of different categories is an ongoing discussion in the scientific world, both in relation with the outcomes of cognitive and emotive trainings, and with analyzing video games. Finally, the follow-up effect of video games training was not specifically addressed in this review, since a very limited number of studies provided follow-up tests.

### Future directions

The present systematic review provides several directions for future studies in this research field. First of all, further studies are needed to better examine the video games effects on cognitive and emotional skills, especially in middle age adults, population which has been investigated in a limited number of studies. Secondly, one of the biggest unresolved issues appears to be the generalizability of improvements: up to now, only short-term effects and specific improvements have been recorded in most studies (e.g., Hardy et al., 2015; Tárrega et al., 2015). In addition, video game characteristics (e.g., genre, platform) in relation with trained skills should be further investigated in the future, in order to create specific and effective training programs.

## Conclusion

To summarize, the present systematic review gives evidences of benefits of video game trainings on cognitive and emotional skills in relation to the healthy adult population, especially on young adults. Efficacy has been demonstrated not only for non-commercial video games or commercial brain-training programs, but for commercial video games as well. As for the distribution of game genre, action games are the most commonly used, followed by puzzle games. Finally, in this review, empirical evidences were identified for all the training outcomes, showing the potential effectiveness of video games for the training of both cognitive (i.e., multiple domain, processing speed and RTs, memory, task-switching/multitasking, mental spatial rotation), and emotional skills.

## Author contributions

FP, AF, and FM conceived the idea of this systematic review. FP and AF examined and write the description of the studies included. FM supervised the scientific asset. FP and AF write the first draft of the paper. All the authors read and approve the final version of the manuscript.

### Conflict of interest statement

The authors declare that the research was conducted in the absence of any commercial or financial relationships that could be construed as a potential conflict of interest.
