# Supplementary material for: Video Games for Well-Being: A Systematic Review on the Application of Computer Games for Cognitive and Emotional Training in the Adult Population
Source: Front Psychol. 2018 Nov 7;9:2127. doi: 10.3389/fpsyg.2018.02127 (PMC6234876; doi:10.3389/fpsyg.2018.02127)
Supplement: Supplementary file 1 [file Table_1.pdf]

Table S1. Information about the selected studies on video games for cognitive training.

Study	Sample	Age	Study Design	Conditions	Time spent playing	Measures	Main outcomes	Effect sizes
Baniqued et al. (2014)	209	18–30 years old	Randomized Controlled Trial	Four conditions:<br>- Working Memory and Reasoning Games Group (WM-REAS)<br>- Adaptive Working Memory and Reasoning Games Group (AWM-REAS)<br>- Active Control Casual Games Group<br>- No-contact Control Group	5 sessions (1- 2 h each) over a median period of 16 days	Recruitment:<br>- Video game players<br>Pre and post training:<br>- Episodic memory and processing speed (Word Recall, Logical Memory, Paired Associates, Digit-symbol Coding, Letter Comparison and Pattern Comparison, Form Boards, Spatial Relations, Paper Folding, Shipley Abstract, Letter Sets, Matrix Reasoning)<br>- Working memory (Visual short-term Memory, N-back, Spatial Working Memory, Running Span, Symmetry Span)<br>- Task-switching (Trial Making, Attentional Blink, Attention Network Test, Color Stroop)	No groups showed gains in working memory, episodic memory or perceptual speed, but the WM-REAS groups improved in task-switching	- Significant Group effect for task-switching ($\eta 2 = 0.092$), with higher gain scores for both WM-REAS training groups<br>- No Group effects were found for episodic memory ($\eta 2= 0.011$), processing speed ($\eta 2= 0.006$), visual attention network ($\eta 2= 0.009$), and working memory ($\eta 2= 0.024$)
Blacker et al. (2014)	34	20.53 ± 2.5 years old	Randomized Controlled Trial	Two conditions:<br>- Action Game Group<br>- Control Game Group	Several sessions (1 h each, 30 h total) over 1 month	Recruitment:<br>- Not having played action game for a year<br>Pre and post training:<br>- Visual working memory (Change Detection; Color Wheel; Symmetry Span)	Visual working memory is increased after an action game training	- Time by Condition interaction was significant on Change detection task ($\eta 2 = 0.16$): only the Action Game Group showed a significant gain in performance from pre to post training (d= 1.82)<br>- Time by Condition interaction did reach significance on Color Wheel ($\eta 2 = 0.16$), with the Action Game Group showing a larger reduction in error after training, relative to the Control Group

Chandra et al. (2016)	5	20 – 27 years old	Randomized Controlled Trial	Two conditions:<br>- Experimental Group<br>- Control Group	Several sessions (1 h each, 50 h total) over 2 months	Pre and post training:<br>- Visual working memory (VISGED)<br>- Processing speed (Determination Test)<br>- Reaction times (Psychology Experiment Building Language - PEBL)	Training improves cognitive abilities like processing speed and reaction time; no effect on visual working memory	- Only the Experimental Group showed a significant decrease in reaction time at PEBL from pre to post training (p= .023) and a significant decrease in Determination Test from pre to post training (p= .005)
Cherney et al. (2014)	60	18 – 22 years old	Randomized Controlled Trial	Three conditions:<br>- Nintendo Wii™ Group<br>- Nintendo GameCube Group<br>- Control Group	One session of 1 h	Recruitment:<br>- Survey on Spatial Representations and activities, and Childhood Activities questionnaire<br>Pre and post training:<br>- Mental spatial rotation (Mental Rotation Test - MRT)	Enhancement of mental spatial rotation abilities after the video game training, with a greater advance for women	- Men significantly outperformed women on the pre-MRT test (d = 0.54)<br>- Women in the video game training condition had significantly higher MRT difference scores than women in the control condition (d =1.20)
Choi et al. (2013)	44	19.28 ± .9 years old	Randomized Controlled Trial	Four conditions:<br>- First Person Shooter (FPS) Group<br>- Third Person Shooter (TPS) Group<br>- Control Puzzle Group<br>- Control Passive Group	30 h total over 10-14 days	Recruitment:<br>- Survey on Spatial Representations and activities, and Childhood Activities questionnaire<br>Pre and post training:<br>- Reaction times (Attentional Visual Field Task – AVF)<br>- Processing speed (Choice Reaction Time - CRT)<br>- Mental spatial rotation (Mental Rotation Task – RT)	Viewpoints of video game may impact differently on Reaction Times and processing speed; however, no significant improvement was found in mental spatial rotation skills, suggesting playing FPS game may not improve dynamic and larger-scaled spatial abilities	- Time by Condition interaction was significant on AVF (η^2= .14): the FPS game group had significant improvements at 20° eccentricity in the AVF, while the TPS game group or the puzzle game group did not show any improvement<br>- Time by Condition interaction was significant on CRT (η^2= .23): the TPS game group decreased processing speed at session 3 and 4, while the FPS game group, the puzzle game group and the control group did not show any significant change over time
Clemenson et al. (2015)	69	18 – 22 years old	Randomized Controlled Trial	Experiment 2<br>Two conditions:<br>- Experimental Group<br>- Active Control Group	One 30 min session per day over 2 weeks	Pre and post training:<br>- Mnemonic discrimination (Recognition Memory Score<br>- MST, Lure Discrimination Index - LDI)<br>- Visual working memory (performance, time spent	Overall improvement in the mnemonic discrimination and spatial memory after the video game training compared to the control condition	- Reliable effect of training on the LDI measure in the Experimental Group (d= 0.86); the effect was reliably larger after Experimental Group than the Control Group (d= 0.499)<br>- Overall improvement in the time spent searching for the platform in the visual working memory in the Experimental Group compared to the

						searching for the platform)		Control Group (d=0.709)
Colzato et al. (2013)	100	21.6 ± 2.7 years old	Quasi-experimental	One condition: Online First Person Shooter (FPS) game	One 30 min session per day over 2 weeks	Recruitment:<br>- Video game questionnaire, DNA laboratory analysis<br>Pre and post training:<br>- Task-switching (Reaction Times - RTs)	Playing FPS games promotes task-switching ability in individuals with a suitable genetic predisposition	- Only Val/Val genotype homozygotes significantly benefited from playing FPS games in RTs, showing decreased switching costs in the post-test compared to the pre-test assessment (d= 1.403)
Dominiak et al. (2016)	12	20-30 years old	Randomized Controlled Trial	Three conditions:<br>- Dance Video Game Group<br>- Shooter Video Game Group<br>- Passive Control Group	6 sessions (1.5 h each, 9 h total) over 3 weeks	Pre and post training:<br>- Mental spatial rotation (MRT-Peters, MRT-Bio, Hidden-Figures-Test)	The training groups show no significant training effects compared to the control group, probably because of the limited number of participants	Statistical significant effect only of Time on MRT-Peters (η^2= .721), and on the Hidden-Figures-Test (η^2= .565)
Green et al.(2012)	18	25.7 ± 0.9 years old	Randomized Controlled Trial	Experiment 4<br>Two conditions:<br>- Action Group<br>- Control Active Group	50 h over the course of several weeks (6–14 weeks)	Recruitment:<br>- Questionnaire related to video game habits<br>Pre and post training:<br>- Reaction times (RTs) and processing speed (Visual Color or Shape Identification Tasks)	The action-trained video game group showed a decrease in reaction times	- The only significant effect on accuracy scores was that of trial type (η^2= .179)<br>- A significant interaction between Test and Group (η^2= .187) indicated that the action Group decreased their RT more between pre-and post-tests than the Control group; an interaction between Group, Test, and Trial Type (η^2=.148) indicated a greater switch cost reduction between pre-and post-tests in the action group as compared to the control group
Hutchinson et al., (2016)	60	18-25 years old	Randomized Controlled Trail	Four conditions:<br>- Training with a FPS game on Microsoft Xbox<br>- Training with a FPS game on Nintendo DS<br>-Training on a visual training game for Nintendo DS	10 sessions, 1 h per day	Pre Post Training:<br>- Reaction Times (RTs) and processing speed for congruent and incongruent targets (Simon Effect)	Significantly faster response times and a reduced cost of stimulus-response incompatibility (Simon Effect) in the groups trained on the FPS; no benefit of training was observed in the control group or the group trained on the	- Time by Congruence interaction was significant ($\eta2$= .146), indicating a general reduction in RTs with training and a larger effect on incongruent compared to congruent trials; no overall effect of Group ($\eta2$= .026)

				- Passive Control Group			visual training game	
Kable et al. (2017)	128	18-35 years old	Randomized Controlled Trial	Two conditions:<br>- Cognitive Training Group<br>- Active Control Group	15 sessions (30 min each), 5 times/week	Pre and post training:<br>- fMRI during performance of validated decision making tasks (delay discounting and risk sensitivity)<br>- Task-switching (Stroop Test, Stop Signal Task, Color Shape Task)<br>- Working memory (Visual/Spatial N-Back Test)	Commercial adaptive cognitive training appears to have no benefits in healthy young adults above those of standard video games for brain activity, choice behavior, or cognitive performance on multiple domains (e.g. task-switching, working memory)	- No significant Condition by Time interaction on decision making tasks (d= 0.05), or on changes in neural activity during choices (d= 0.19)<br>- Participants in both groups improved in cognitive performance examining composite cognitive scores (d= 2.37); however there was no significant Condition by Time interaction (d= 0.19)<br>- Participants in both groups improved in Stroop Test (d= 0.36), Stop Signal Task (d= 1.71), Color Shape Task (d= 0.54), Visual/Spatial N-Back Test (d= 1.308); no significant Condition by Time interaction on any of these
Kuhn et al. (2014)	48	24.1 ± 3.8 years old	Randomized Controlled Trial	Two conditions:<br>- Video Game Training Group<br>- Passive Control Group	30 min per day over 2 months	Recruitment:<br>- No video game usage in the past 6 months<br>Pre and post training:<br>- fMRI while playing a tunnel task to assess spatial orientation preferences, and working memory	Video game training augments gray matter in brain areas crucial for spatial navigation, working memory and motor performance, going along with evidence for behavioral changes of navigation strategy	- The gray matter volume in dorsolateral prefrontal cortex shows a clear increase in the Training group (d= 0.667), and a significant difference between both groups at post-test (d= 0.667)<br>- The habenular commissure (d= 0.852), and the cerebellum (d= 0.609) differed significantly at post-test
Lee et al. (2012)	75	18–30 years old	Randomized Controlled Trial	Two conditions:<br>- Hybrid Variable-Priority Training (HVT)<br>- Full Emphasis Training (FET)	15 sessions (2 h each), 3-5 sessions a week, over 8–10 weeks	Pre post training:<br>- Working memory (Sternberg Memory Task, N-Back memory Task)<br>- Reaction times and processing speed (Attention Blink Task, Dot Comparison Task, Change Detection Task, Flanker Task, Manual Sequence Task – accuracy and RTs)<br>- Dual-tasking ability	Task-specific improvements after the training in WM, which do not transfer from trained to untrained tasks (reaction times and processing speed, dual-tasking ability, etc.)	- After 10 h of training the contrast between HVT and FET was not significant in all tasks except the Sternberg Memory Task, favoring HVT over FET (p= .014);<br>- After 30 h of training the contrast between HVT and FET was not significant in all tasks (p> .05)

						(Dual Task Manual Control, Radar monitoring Task		
Li et al. (2016)	12	19–37 years old	Randomized Controlled Trial	Experiment 3<br>Two conditions:<br>- Video Game Group<br>- Active Control Group	10 sessions (1 h each), 1 or 2 session per day over 3 weeks	Pre, middle (5hr), and post (10hr) of training:<br>- Reaction times and processing speed (Visuo-motor Control Task)	Improvements in the reaction times and processing speed only after the video game training	- Significant interaction of Condition and Test session ($\eta 2= .41$): whereas the Action Group's mean response gain increased by 24% after 5 hr of play ($p = .0012$) and by 30% after 10 hr of play ($p = .00011$), compared with the group's pretest performance, the Control Group's mean response gain did not increase significantly after 5 or 10 hr of play
Looi et al. (2016)	30	24.2 ± 2.1 years old	Randomized Controlled Trial (three conditions)	Three conditions:<br>- Real tDCS during mathematics Video Game Training<br>- Sham tDCS during mathematics Video Game Training<br>- Active Control Group	2 sessions on 2 separate days (within 3 days)	Recruitment: Standardized mathematics test (WIAT-II UK)<br>Pre and post training:<br>- Working memory (Digit Span, Corsi Blocks)	Effects to working memory in both the video game groups, and such effects remained partially until two months post-training	- Verbal WM: interaction between Time and Condition ($\eta 2 = .37$): tDCS and Sham group did not differ in capacity pre-test (d= 0.63), but at post-test the tDCS group showed an improved verbal WM capacity (d= 0.72), while shame group performance did not change<br>- Visuospatial WM: interaction Time x Group no significant<br>- Two months after training, the tDCS group showed a sustained effect in verbal WM capacity (d= 0.63), but no effect in visuospatial capacity
Mathewson et al. (2012)	39	18–28 years old	Quasi-experimental	One condition: Space Fortress game	10 sessions (2 h each, 20 h total)	Recruitment: Having played fewer than 3 h of video games a week in the past 2 years<br>Pre and post training:<br>- Alpha power, event-related spectral perturbations (ERSPs), and event-related brain potentials during early training of the Space Fortress task were recorded<br>- Battery of cognitive tasks, testing task-switching, and working memory (Task Switching,	Control processes, as indexed by alpha and delta oscillations, can predict learning and improvements in WM but not on task-switching	- Greater fortress-locked delta ERSPs power was predictive of reductions in response times in the Sternberg memory search task ($p< .05$), and in the Stop Task ($p< .05$); reduced alpha suppression following fortress hits predicted decreases in both Sternberg response time ($p< .05$), and focus switch costs in the N-back task ($p< .05$)<br>- Baseline levels of alpha power significantly predicted only changes in task switching (all $p< .05$); however, reductions in task switching costs were not significantly related to any of the ERSPs measure

						Stopping Task, Sternberg Memory Test, N-Back Task)		
Montani et al. (2014)	20	19–25 years old	Quasi-experimental	One condition:<br>- A custom-made video game including the Diamond Task and the Snake Task	40 min per day over 2 weeks	Pre and post training:<br>- Task-switching and dual-tasking (DT)	The cost of dual tasking as well as the cost of task switching decreased after the video game training	- The decrease in DT was significant for both conditions (d= 1.54), but the reduction was larger for the dual task condition<br>- Task Switch effect: the interaction Session by Condition was significant (d=1.91) indicating that the effect of the session was different for the two conditions: the decrease DT was significant for both conditions, but the reduction was larger for the switch (new task) condition
Nikolaidis et al. (2014)	45	21.7 ± =5.1 years old	Quasi-experimental	One condition:<br>- Space Fortress game	15 sessions (2 h each, 30 h total)	Recruitment:<br>- Having played videogames less than 4 h per week<br>Pre and post training:<br>- Working memory (Sternberg Memory Search – SMS; Change Detection - CD)<br>- Functional magnetic resonance imaging (fMRI) scans while they played the training videogame	For regions implicated in working memory, individual differences in the post-minus-pre changes in activation predicted performance changes in an untrained working memory task: training-induced plasticity in the functional representation of a training task may play a role in individual differences in transfer	- Changes in percent signal change predicted 32% of the variance in the performance changes to the SMS task (d= .605)

Nouchi et al. (2013)	41	20.7 ± 1.2 years old	Randomized Controlled Trial	Two conditions:<br>- Brain Age Training Group<br>- Active Control Group	At least 5 sessions a week (15 min each) over 4 weeks	Recruitment:<br>- Non-gamers (reported playing video games less than one hour of a week over the prior 2 years)<br>Pre and post training:<br>- Task-switching (Wisconsin Card Sorting Test – WCST, Stroop Task - ST)<br>- Working memory (Operation Span - OpS, Letter–Number Sequence - LNS, Arithmetic –Ari)<br>- Short term memory (Digit Span - DS, Spatial Span -SpS)<br>- Reaction times (RTs) and processing speed (Attention Digit Cancellation Task - D-CAT, Simple Reaction Time – SRT, Digit Symbol Coding – Cd, Symbol Search - SS)<br>- Mental spatial rotation (Mental Rotation Task - MR)	Commercial brain training improves task-switching, working memory Reaction Times (RTs), and processing speed more than the selected puzzle game	- Results showed that the Brain Age group improved all measures of the executive functions (WCST, $\eta 2= 0.23$; ST, $\eta 2= 0.25$¸rST , $\eta 2= 0.20$), all measures of the WM (OpS, $\eta 2= 0.15$; LNS, $\eta 2= 0.14$; Ari, $\eta 2= 0.21$), and all measures of the RTs and processing speed (Cd, $\eta 2 = 0.23$; SS, $\eta 2= 0.24$) compared to the Tetris group<br>- The Tetris group improved one of two measures of RTs and processing speed (SRT, $\eta 2$ 0.10) and the measure of mental spatial rotation ability (MR, $\eta 2= 0.18$) compared to the Brain Age group<br>- Playing Brain Age or Tetris did not improve one of two measures of RTs and processing speed (D-CAT, $\eta 2= 0.00$), any measure of short term memory (DS-F. $\eta 2= 0.00$; DS-B, $\eta 2= 0.00$; SpS-F, $\eta 2= 0.00$; SpS-B, $\eta 2= 0.00$)
Oei et al. (2013)	75	21.07 ± 2.1 years old	Randomized Controlled Trial (four conditions)	Four conditions:<br>- Action Video Game Group<br>- Hidden-object Game Group<br>- Match-3 Group<br>- Memory Matrix Group	5 sessions a week (1 h each, 20 h total)	Pre and post training:<br>- Reaction times and processing speed (Attentional Blink Task)<br>- Spatial working memory (Visual Search Dual Task)	Improvement in Reaction Times (RTs) and processing speed was not limited to the action video games, as each game resulted in an enhanced performance in the tasks which shared common demands	- Attentional Blink Task RTs: only the Action Group showed a statistically significant improvement on the target 2 (d= -2.19), target 3 (d= -2.26) and the target 4 (d= -1.53)<br>- Visual Search processing speed: improvement from pre to post training in the dual task condition for the Match-3 group was significant for set-size 4 (d= -1.32), set size 8 (d= -2.81) and set size 12 (d= -1.35)<br>- Visual Search RT: the hidden-object game group had a significant reduction in RT for all set sizes in both single and dual task conditions (search-only condition of set size 4, d =1.54, set size 8 (d= 0.91), and set

								size 12 (d= 0.85); dual-task condition of set size 4 (d= 2.49); set size 8 (d= 1.81); set size 12 d= 1.91), the memory matrix game group had a reduction in RT in set size 4 (d= 1.23), and set size 8 (d= 1.21), for the search-only conditions.; for the match-3 group, there were significant RT improvements seen in search-only set size 8 (d= 1.21), and set size 12 (d= 1.02); for the dual-task condition, there was a reduction in RT for set size (d= 1.34)<br>- Spatial Memory Test Accuracy: the hidden-object group improved in the dual-task condition, set sizes 8 (d= -3.13) as well as set size 12 (d= -1-21)
Oei et al. (2014)	52	19 – 24 years old	Randomized Controlled Trial (four conditions)	Four conditions:<br>- Action Video Game Group<br>- Puzzle Game Group<br>- Real-time Strategy Game Group<br>- Arcade Game Group	5 sessions a week (1 h each, 20 h total	Recruitment:<br>- No regular video game players (less than one hour per week for the previous year)<br>Pre and post training:<br>- Task-switching (Random Task Switching, Flanker, Response Inhibition Task Go/No-go)	Only the group that trained on the physics-based puzzle game significantly improved in all three tasks: playing a complex puzzle game that demands strategizing, reframing, and planning improves task-switching	- Task Switching: the Modern Combat (d= .47), Fruit Ninja (d= .47), and Starfront Collision groups (d= .777) had no significant reduction in switch cost from pre- to post-training; the Cut the Rope group significantly reduced switch cost as a result of training (d= 1.31)<br>- Go/no Go: the Modern Combat (d= .50), Fruit Ninja (d= .10) and Starfront Collision (d= .31) groups failed to significantly reduce false alarm rates from pre- to post-training; the Cut the Rope group had a significant reduction in false alarm rate from pre- to post- training (d= 1.19)
Oei et al. (2015)	54	21.78 ± 1.7 years old	Randomized Controlled Trial (four conditions)	Four conditions:<br>- Metal Gear Solid Touch (MGS) Group<br>- Modern Combat Group<br>- Super Sniper Group<br>- Deer Hunter Group	5 days a week (1 h each, 20 h total)	Pre and post training:<br>- Reaction times and processing speed (Attentional Blink; Filter Task)<br>- Visual working memory (Visual Search)	Only the Modern Combat group improved in the ability to apprehend multiple objects simultaneously enhancing RTs and processing speed	- Attentional Blink: improvement by the Modern Combat group in accuracies for lag 2 (d=1.61), lag 3 (d=1.43), lag 4 (d=0.95), and lag 5 (d=1.00); statistically significant improvements in lag 3 (d=1.46), lag 4 (d=1.76), and lag 5 (d=1.22) for the MGS Touch group; aside from the Super Sniper group's improvement in lag 5 (d=1.05), no other improvements were seen;

								- Filter Task: for the Modern Combat group, improvement from pre- to post- training for the 2 target 6 distractor condition approached significance (d=0.84);<br>- Visual Search Accuracy and RTs: the groups did not differ in the magnitude of improvements
Parong et al. (2017)	Study 1: 45	18–24 years old	Randomized Controlled Trial	Two conditions:<br>- Experimental Group<br>- Active Control Group	4 sessions (30-minutes each, 2 h total) over a period of 2 weeks	Pre and post-training:<br>- Task-switching (Dimensional Change Card Sort test - DCCS, and Letter-Number task - LN)	The experimental group developed significantly better performance on cognitive shifting tests compared to the control group after playing for 2 hours over 4 sessions (i.e., reaching a high level in the game), but not when they played for 1 h over 2 sessions	- The Alien Game Group performed significantly better than the Bookworm Group on the LN task (d= 0.82), but the difference did not reach statistical significance on the DCCS (d= 0.08)<br>- The Alien Game group achieved a significantly higher composite shifting score on the post- test than the Bookworm Group (d= 0.54)
	Study 2: 49	18–35 years old	Randomized Controlled Trial	Two conditions:<br>- Experimental Group<br>- Active Control Group	2 sessions (30-minutes each, 1 hour total) over a period of 1 week	Pre and post-training:<br>- Task-switching (Dimensional Change Card Sort test - DCCS, and Letter-Number task - LN)		- The Alien Game Group and the Bookworm Group did not differ significantly on the DCCS (d= 0.41) or the LN task (d= 0.07)
	Study 3: 89	18–24 years old	Randomized Controlled Trial	Three conditions:<br>- No Goal Group<br>- Goals Group<br>- Control Passive Group	4 sessions (30-minutes each, 2 hours total) over a period of 2 weeks	Pre and post-training:<br>- Task-switching (Dimensional Change Card Sort test - DCCS, and Letter-Number task - LN)	Results show the effectiveness of playing a custom-made game that focuses on a specific executive function skill for sufficient time at an appropriate level of challenge; practice of a cognitive skill in a game context is transferred to performance on the same skill in a non-game context	- No significant difference between the Goal Group and No Goal Group on mean post-test scores for the DCCS task (p= 0.080), the LN task (p= 0.581), or for a composite score computed (p= 0.501);<br>- The Game Group (i.e., the combined Goal and No Goal Groups) achieved significantly higher post-test scores than the Control Group on the DCCS (d= 0.75), and on the composite score (d= 0.78);<br>- The Game Group did not achieve significantly higher scores than the Control Group (d= 0.45)

Rolle et al. (2017)	42 young adults 40 older adults	Young, 23.6 ± 2.8 years old<br><br>Older, 67.3 ± 4.5 years old	Randomized Controlled Trial (four conditions)	Four conditions:<br>- Training Young Adults<br>- Control Young Adults<br>- Training Older Adults<br>- Control Older Adults	5 sessions per week (30-min each, 5 h total) over 2 weeks	Recruitment:<br>Mini mental State Examination (minimum 26); assessment of memory, executive functions, and motor evaluation<br>Pre and post training:<br>- Reaction times (RTs)<br>- Spatial working memory (CDT)	Video game training led to improved focused and distributed attention abilities as well as improved spatial working memory and spatial attention in both younger and older participants	- The Time × Study group interaction was significant on RT (η2= .069); the Train Groups significantly enhanced their RT performance from pre-training to post-training on all Cue information levels, whereas the Control Group did not improve<br>- Time × Study group interaction was significant on CDT (η2=.096)
Shubert et al. (2015)	Study 2: 62	25 ± 3.5 years old	Quasi-experimental	Three conditions:<br>- Action Video Game Group<br>- Puzzle Video Game Group<br>- No-contact Group	15 sessions (1 h each) over 4 weeks	Recruiting:<br>- No video-game practice in the 6 months prior to testing<br>Pre and post training:<br>- Processing speed<br>- Visual working memory	No effects of action video gaming on visual WM, and processing speed resulted from training; however, observations of a selected improvement of processing speed after video game training are suggestive for limited possibilities to improve basic aspects of visual attention with practice	- Visual threshold: no main effects of group<br>(η2=.05), as well as no significant interaction (η2= .04)<br>- Visual perceptual processing speed increased significantly from pre- to post- test (η2= .29); no significant effect of group or interaction including group<br>- Short-term storage capacity generally increased from pre-test to post-test (η2= .09); this increase was the same for all three groups<br>- The three groups did not differ in the size of the iconic memory buffer, nor are there any effects of session and of the interaction between session and group (η2= .03)
Shute et al. (2015)	77	18–22 years old	Randomized Controlled Trial	Two conditions:<br>- Commercial Game Group;<br>- Brain Training Game Group	4 sessions across 1 to 2 weeks (for a total of 8 hours)	Pre and Post-training:<br>- Mental spatial rotation (Mental Rotation Test - MRT, Spatial Orientation Test - SOT, Virtual Spatial Navigation Assessment -VSNA)	Participants who were assigned to play Portal 2 showed a statistically significant improvement in mental rotation skills, while there were no significant pretest-to-posttest improvements in any of the three spatial test for	- Participants in the Portal 2 condition showed pre-test to post- test improvement on the MRT (d = .30); Portal 2 players' VSNA scores significant differed from pretest-to-posttest (d = .44); no significant improvement for Portal 2 players on the SOT test; for participants in the Lumosity condition, there were no significant pretest-to-posttest improvements on any of the three spatial tests

							participants in the Lumosity condition	
Stroud et al. (2015)	Study 2: 58	Mean age =19.57	Randomized Controlled Trial	Three conditions:<br>- 0 rounds of Bejeweled (BJB)<br>- Playing 10 rounds of BJB<br>- Playing 30 rounds of BJB	One session (0, 10, or 30 rounds of 1 min each)	Post training:<br>- Reaction times and processing speed (visual search stimuli)	Reaction times were significantly faster for participants who completed 30 rounds compared with the search task only	- BJB scores did not significantly improve for participants completing only 10 training rounds of BJB, but scores did significantly improve for participants playing 30 training rounds ($p < .027$)<br>- RTs were significantly faster in the 30 training round condition than in the 10 training round condition for both target absent and target present trials ($p < .015$)
	Study 3: 59	18–31 years old<br>(M =21<br>SD = 1.8)	Randomized Controlled Trial	Three conditions:<br>- 0 rounds of BJB<br>- Playing 10 rounds of BJB<br>- Playing 30 rounds of BJB	One session (0, 10, or 30 rounds of 1 min each)	Post training:<br>- Reaction times (RTs) and processing speed (visual search stimuli)	Numerical but not significant trend similar to previous results	- Scores significantly improved across 30 rounds (p =- 012)<br>- RTs performance improved across target absent trials across the three Training Rounds, but there was no significant interaction between Training Round (p= .30)
van Ravenzwaaij et al. (2014)	Study 1: 20	20.6 ± 2.4 years old	Randomized Controlled Trial	Two conditions:<br>- Action Video Game Condition<br>- Cognitive Video Game Condition	5 sessions (2 hours each, 10 h total) with 10 mins	Pre and post training:<br>- Reaction times (RTs) and processing speed (Perceptual Discrimination Task)	Decreased mean RT for the action game compared with playing the cognitive video game; response accuracy decreased slightly over sessions, hinting at the possibility that participants became less cautious as they improved with practice	- Negative linear trend over sessions on mean RT (d=3.43); this session effect for mean RT did not interact with gaming condition (d= .24).<br>- Participants made more mistakes in subsequent sessions across conditions (d= 1.21); no evidence for an interaction between session and gaming condition for accuracy (d=.6)

	Study 2: 45	20 ± 1.8 years old	Randomized Controlled Trial	Three conditions:<br>- Action Video Game Group<br>- Cognitive Video Game Group<br>- Passive Control Group	5 sessions (4 hours each, 20 h total) with three self-paced breaks after each hour	Pre and post training:<br>- Reaction times (RTs) and processing speed (Perceptual Discrimination Task)	Faster performance for the non-game condition and the cognitive game condition, but not for the action game condition.	- Across conditions participants' mean RTs shortened in subsequent sessions ($p < .001$); as the number of sessions increased, participants speeded up both in the cognitive game condition, ($p < .001$), and in the no-game condition ($p < .01$), whereas they did not speed up in the action game condition, ($p > .05$)<br>- Significant positive linear trend over sessions for accuracy ($p < .001$); there was no evidence for an interaction between session and gaming condition for accuracy ($p > .05$)
Wang et al. (2014)	30	18-25 years old	Quasi-experimental	Four conditions:<br>- Young video-game players (VGP)<br>- Young non-video-game players (NVGP)<br>- Old VGP<br>- Old NVGP	5 sessions (~1.5 hour each) over four weeks	Recruitment:<br>- Amount of hours played during the previous 6 months<br>Pre- Post-training:<br>- Reaction times (RTs) and processing speed (Flanker Task)	A training effect was found for the younger adults but not for older adults, therefore playing action-video games can influence attention resources after a short training, at least for young adults	- RTs: the Group main effect was not significant ($p = .49$)<br>- Flanker (accuracy): the effect of Group was significant, which indicated that older NVGPs were significantly less accurate than young VGPs ($p < .05$), and the young NVGPs was in the middle
Wu and Spence (2013)	Study 2: 60	18-25 years old	Randomized Controlled Trial	Three conditions:<br>- FPS Game Group<br>- Driving-racing Game Group<br>- Active Control Group	Several sessions (1 or 2 h each, 10 h total) over 3 weeks	Pre and post-training assessment:<br>- Reaction times (RTs) and processing speed (Classic Dual Search Task)	Participants who played an action or a driving-racing game achieved greater gains on RTs and processing speed than did those who trained using the puzzle game	- Players were more accurate than non-players in the peripheral component of the dual search ($d = .597$); players were also more accurate than non-players in the central component of the dual search ($d = 0.571$), but were not more accurate than non-players in the peripheral search ($d = .34$)<br>- Speed: in the peripheral component of the dual search players were faster than non-players ($d = 0.705$); in the peripheral search alone, players were faster than non-players ($d = -726$)
